# The dyadic paradox of unmet supportive care needs and depression in colorectal cancer couples: an explanatory sequential mixed-methods study

**DOI:** 10.3389/fpsyg.2026.1831478

**Published:** 2026-06-08

**Authors:** Yuan Ruan, Wenhua Zhang, Chengcheng Zhu, Jie Zhao, Zheng Sun

**Affiliations:** 1 Affiliated Hospital of Jiangsu University, Zhenjiang, China; 2 Affiliated Hospital of Jiangnan University, Wuxi, China

**Keywords:** colorectal cancer, depression, dyads, mixed-method study, supportive care needs

## Abstract

**Purpose:**

Guided by the Dyadic Coping Theory (DCT), this study aimed to investigate dyadic interrelationships between unmet supportive care needs (USCNs) and depression in colorectal cancer (CRC) patient-spousal caregiver dyads.

**Methods:**

Using an explanatory sequential mixed-methods design, 200 CRC patient-caregiver dyads completed quantitative surveys. The Actor-Partner Interdependence Model (APIM) analyzed dyadic data. Subsequently, 10 dyads participated in semi-structured interviews, analyzed using reflexive thematic analysis.

**Results:**

Patients reported significantly higher USCNs and depression. APIM revealed significant actor effects (unfulfilled needs predicted intrapersonal depression) but non-significant partner effects. Qualitative findings explained this phenomenon: couples employed “protective buffering” to conceal unfulfilled needs, inadvertently causing emotional isolation.

**Conclusion:**

The interrelationship between USCNs and depression in CRC dyads is driven by intrapersonal internalization rather than direct interpersonal transmission. Mutual protective buffering hinders open communication rather than directly transmitting distress. Care must transition to a dyad-centered approach, implementing couple-based communication interventions to dismantle protective buffering and alleviate mutual distress.

## 1 Introduction

The International Agency for Research on Cancer (IARC) reported that colorectal cancer (CRC) ranked third in incidence and second in mortality globally in the Global Cancer Statistics 2022 (Bray et al., 2024), posing a major global public health challenge. Beyond physical symptoms such as diarrhea and constipation, CRC patients face substantial psychological distress, notably depressive symptoms (Dunn et al., 2013). This distress is shared with their spousal caregivers, who serve as the patients’ primary physical and emotional supporters (Grov et al., 2005). Consequently, the trajectory of CRC impairs the health and wellbeing of the entire dyad, generating diverse unmet supportive care needs (USCNs) and exacerbating mutual depression (Niu et al., 2021).

However, existing studies on the relationship between USCNs and depression have yielded inconsistent results. While some indicate that higher USCNs correlate with more severe depression, others report an inverse association (Sklenarova et al., 2015). These inconsistencies underscore significant methodological and conceptual limitations in current research. These inconsistencies suggest that the association between USCNs and depression may not be purely individual or linear, but shaped by dyadic interdependence and culturally embedded protective behaviors (Qiu et al., 2025a). Although prior dyadic studies have advanced understanding of reciprocal influence and shared adjustment within cancer couples, they have often focused on broad psychosocial outcomes rather than the specific pathway linking USCNs to depressive symptoms (Sun et al., 2025).

First, most studies adopt an individualistic perspective, focusing solely on either patients or caregivers, thereby neglecting the dyad as an interactive emotional unit (Sun et al., 2024). Second, the majority of research relies heavily on purely quantitative designs. While quantitative studies can identify correlations, they fall short in capturing the nuanced, lived experiences of USCNs and the complex communication dynamics between partners (So et al., 2019). In the Chinese context, familism, relational harmony, and spousal responsibility may shape how CRC dyads express and manage USCNs (Wu et al., 2022). Rather than openly disclosing distress, patients and caregivers may withhold emotional or informational needs to protect one another, which may complicate the association between USCNs and depression (Qiu et al., 2025a).

To address these gaps, this study is grounded in Dyadic Coping Theory (DCT). DCT posits that couples function as an interdependent system in appraising and coping with the chronic stressors of cancer (Wenhai and Mingming, 2023). Couples may employ positive dyadic coping (e.g., joint problem-solving and open emotional expression) to foster resilience, or negative dyadic coping (e.g., mutual avoidance, emotional concealment, and protective buffering) which often leads to emotional isolation (Xuehua et al., 2025; Falconier and Kuhn, 2019). Understanding how CRC dyads utilize these coping strategies is essential for deciphering the complex interrelationship between their unfulfilled needs and depressive symptoms. However, little is known about the interactive relationship between USCNs and depression from a dyadic perspective in CRC dyads (Hu et al., 2022).

To effectively capture the interdependent nature of these dyads, this study utilized the Actor-Partner Interdependence Model (APIM) proposed by Kenny and Ledermann (2010). By nesting individual measurements within dyads, APIM accounts for the non-independence of dyadic data, allowing for the simultaneous estimation of “actor effects” (the impact of one’s own USCNs on their own depression) and “partner effects” (the cross-over impact of one’s USCNs on the partner’s depression) (Qiu et al., 2025a; Kim and Lee, 2023). However, APIM can identify whether actor and partner effects exist, but it cannot explain why such dyadic patterns occur. Therefore, the explanatory sequential mixed-methods design allowed us to test the USCNs–depression associations quantitatively and then use qualitative interviews to explain how CRC dyads concealed, internalized, or shared USCNs, thereby explaining the psychosocial mechanisms underlying the APIM findings (Kawar et al., 2024).

By integrating DCT with APIM through a mixed-methods approach, this study aimed to: (1) describe and compare the profiles of USCNs and depression in CRC patient-spousal caregiver dyads; (2) quantitatively examine the actor and partner effects between patients’ and caregivers’ USCNs and depression using the APIM; and (3) qualitatively elucidate the underlying psychosocial mechanisms driving these dyadic patterns, thereby exploring more targeted, couple-based interventions.

## 2 Methods

### 2.1 Study design

An explanatory sequential mixed-methods design was employed, which consisted of two distinct phases: an initial quantitative phase followed by a subsequent qualitative phase (Kawar et al., 2024). The quantitative phase aimed to identify the perceived USCNs and levels of depression among CRC patient-caregiver dyads, and to examine the relationship between depression and dyadic USCNs. In the qualitative phase, semi-structured interviews were conducted to explore the specific USCNs of CRC dyads in depth and to further investigate the survey responses obtained during the quantitative phase. In this explanatory sequential design, the quantitative results guided the purposive sampling for the qualitative phase to identify CRC dyads with representative interaction patterns and varying levels of USCNs. Furthermore, these quantitative findings informed the development of the semi-structured interview guide. The quantitative and qualitative strands were integrated at the interpretation stage to explain how the qualitative themes contextualized the APIM findings. All procedures were approved by the Research Ethics Committee of Jiangnan University Hospital (LS2023060).

### 2.2 Participants and setting

Between August 2024 and May 2025, CRC patient-spousal caregivers were recruited through convenience sampling from a tertiary hospital in Wuxi, China. Eligible participants had to meet the following criteria: (a) be married Chinese adult dyads; (b) one spouse diagnosed with CRC; (c) the other spouse serving as the primary caregiver, responsible for the majority of caregiving tasks; and (d) both spouses voluntarily agreeing to participate and able to communicate in Mandarin. Couples were excluded if either individual had a severe psychiatric disorder or communication impairment that precluded their participation. *A priori* power analysis was conducted using G*Power 3.1 for a Pearson correlation test. With parameters set to a small-to-moderate effect size (*r* = 0.20), a two-tailed *α* of 0.05, and a desired power of 80% (Qiu et al., 2025b), the analysis indicated a minimum sample size of 199 dyads. In the qualitative phase, we employed a maximum variation sampling strategy to purposively recruit CRC dyads from those who had completed the quantitative phase (So et al., 2019). This approach aimed to capture a wide spectrum of experiences by selecting dyads who reported varying levels of USCNs, different degrees of depression, and diverse sociodemographic backgrounds. Data collection and preliminary analytic engagement proceeded iteratively, enabling the researchers to assess the richness, relevance, and interpretive depth of participants’ accounts in relation to the qualitative aim.

During the quantitative phase, data were collected by researchers who received consistent training. Before the survey, potential participants were informed about the study’s goal, purpose, and relevance, and questionnaires were delivered after they provided informed consent. Both CRC dyads completed the surveys independently, with researchers offering advice based on standardized terminology learned during their training. Initially, 220 patient-caregiver dyads were recruited. However, 15 patients declined due to busyness, and another 5 refused for health reasons, resulting in a final sample of 200 dyads and a 90.9% total response rate. Demographic and disease-related characteristics were collected from the CRC dyads. The data included age, gender, educational level, employment status, time since diagnosis, duration of caregiving, and perceived family financial burden attributed to the disease.

The USCNs of the dyads were assessed using two validated instruments. The Chinese version of the Cancer Survivors’ Unmet Needs (CaSUN) scale was used for patients (Molassiotis et al., 2017). This 35-item instrument demonstrated good internal consistency in the current sample (Cronbach’s *α* = 0.846) and retained its original five-factor structure (Li et al., 2020b). For spousal caregivers, the Chinese version of the Cancer Survivors’ Partners’ Unmet Needs (CaSPUN) scale was administered (Hodgkinson et al., 2007). It also showed acceptable internal consistency (Cronbach’s α = 0.752) and comprises 35 items across four factors (Li et al., 2020a). Both scales are rated on a 5-point Likert scale, from 0 (“No Need”) to 4 (“High Need”), with higher scores indicating greater USCNs (Li et al., 2020a). These instruments were selected because patients and spousal caregivers experience distinct but related USCNs, and previous dyadic research has recommended their combined use in survivor–caregiver dyads (Li et al., 2020a). Given their shared 35-item structure, 5-point response format, and same total score range, total scores were used as role-specific indicators of overall USCNs burden in between-member comparisons and APIM analyses (Qiu et al., 2025a). Because the two instruments differ in factor structure, subscale-level comparisons were not conducted.

Depression was evaluated using the well-validated Chinese version of the Hospital Anxiety and Depression Scale (HADS), specifically its depression subscale. The subscale demonstrated excellent internal consistency for both patients (Cronbach’s *α* = 0.917) and caregivers (Cronbach’s α = 0.839) (Li et al., 2016). It contains 7 items, each scored from 0 to 3, where 0 indicates the absence of a problem, and 3 represents a severe problem. Higher total scores signify more severe psychological distress.

During the qualitative phase, the interview guide was specifically refined based on the initial quantitative results, in accordance with the explanatory sequential design. Because the quantitative analysis identified distinct patterns regarding how patients’ and caregivers’ USCNs influenced their own and their partner’s depression, the semi-structured interviews were designed to explore the underlying mechanisms of these dyadic patterns. Specifically, the interviews aimed to explore: (1) how individuals processed their own unfulfilled needs; (2) the specific dyadic interaction patterns and communication strategies couples used to manage mutual psychological distress; and (3) the lived experiences of these emotional boundaries within the context of cancer care.

### 2.3 Data analysis

Quantitative data were analyzed using SPSS 26.0. Quantitative data are presented as mean ± standard deviation (x ± s), and categorical data are reported using frequency and percentage (%). Data completeness exceeded 99% across all core study variables. Little’s Missing Completely at Random (MCAR) test indicated that the missing data were consistent with MCAR (*p* > 0.05). Given the very small proportion of missing data, listwise deletion was applied in the quantitative analyses.

A paired samples *t*-test was employed to analyze differences in USCNs and depression scores among CRC dyads. The Pearson correlation coefficient was used to assess the relationship between USCNs and depression scores in these dyads. Prior to constructing the APIM, the non-independence of the dyadic data was assessed by calculating the Intraclass Correlation Coefficient (ICC) derived from an intercept-only null model. According to Kenny and Ledermann (2010), an ICC > 0.05 warrants the use of APIM analysis. An overall test of distinguishability was also conducted to validate the two-intercept structural model specification.

The APIM was estimated using multilevel modeling (MLM) for distinguishable dyads. Overall USCNs were entered as predictor variables and depression scores as outcome variables. All predictor variables were grand-mean centered before APIM estimation to retain between-dyad variation in USCNs and to make the model intercepts interpretable as expected depression scores at the sample’s average level of USCNs. Unstandardized estimates (b), standardized estimates (β), and *p*-values were reported. Statistical significance was set at a two-tailed α = 0.05.

For the qualitative phase, audio recordings were transcribed verbatim and analyzed using reflexive thematic analysis (RTA) to *develop* themes that *contextualized* the quantitative APIM findings (Braun and Clarke, 2006). RTA was selected because it supports an interpretive and reflexive analysis of how participants make sense of their experiences, making it suitable for explaining the quantitative APIM findings through CRC dyads’ accounts of USCNs and depressive symptoms (Braun and Clarke, 2006). DCT informed the interview guide, but it was not used as a predetermined coding framework or as a template for theme development. The analysis was primarily inductive within a reflexive thematic analysis approach, with coding and theme development conducted through researchers’ reflexive engagement with participants’ accounts, following Braun and Clarke’s six-phase process (Braun and Clarke, 2006). After repeated reading and analytic note-taking, initial codes were generated across the dataset, focusing on both explicit statements and latent meanings related to USCNs, depression, and dyadic experiences. Codes were then collated into candidate themes, which were reviewed, refined, defined, and named through reflexive team discussions. Finally, the themes were integrated with the APIM findings to explain the observed dyadic patterns. To enhance methodological integration, we adopted a ‘joint display’ approach, formally linking quantitative results with qualitative findings in order to assess the extent of their consistency and divergence.

## 3 Results

### 3.1 General characteristics of CRC patient-spousal caregiver dyads

In the quantitative study, participants had a mean age exceeding 64.0 years. The proportion of male CRC patients (67.0%) was higher than that of female CRC patients (33.0%). Most dyads reported a low level of educational attainment, with middle school or below completed by 64.0% of patients and 65.0% of spousal caregivers. The majority were also unemployed (77.0% of patients and 78.0% of spousal caregivers). The time since diagnosis varied widely, ranging from 1 to 92 months. More than half (55.0%) of the spousal caregivers had provided care for less than 6 months. As a result of CRC treatment, 95.0% of families reported facing varying degrees of financial hardship.

The qualitative phase included 10 dyads. Among the patients (P1-P10), 70% were male, with ages ranging from 32 to 68 years. In terms of the education level, 60% had high school or higher degrees. The spousal caregivers (C1-C10) were aged 30–70 years. Regarding the level of education, 50% had completed high school or above. Comprehensive participant characteristics are summarized in Table 1.

**Table 1:** Descriptive characteristics of colorectal cancer patients and spousal caregivers (*n* = 200 dyads).

**Characteristics**	**CRC patients *n* (%)**	**Spousal caregivers *n* (%)**
Age (years)		
Mean	64.3	64.6
SD	10.4 (ranging from 33 to 82)	10.3 (ranging from 34 to 82)
Gender		
Male	134 (67.0)	66 (33.0)
Female	66 (33.0)	134 (67.0)
Level of education		
Middle school or less	128 (64.0)	130 (65.0)
High school	64 (32.0)	66 (33.0)
University and above	8 (4.0)	4 (2.0)
Working status		
Working	46 (23.0)	44 (22.0)
Not working	154 (77.0)	156 (78.0)
Economic burden caused by the treatment		
Severe	64 (32.0)	50 (25.0)
Normal	100 (50.0)	98 (49.0)
Mild	26 (13.0)	43 (21.5)
No	10 (5.0)	9 (4.5)
Duration from diagnosis (months)		
Mean	15.63	
SD	18.2 (ranging from 1 to 92)	
Care-giving time		
Less than 6 month		110 (55.0)
6 to 24 months		52 (26.0)
More than 24 months		38 (19.0)
Changes in marital relationships following illness		
Improve	8 (4.0)	11 (5.5)
No change	72 (36.0)	75 (37.5)
Decline	120 (60.0)	114 (57.0)

CRC, colorectal cancer; SD, standard deviation.

### 3.2 Descriptive statistics and bivariate correlations

For CRC patients, the mean total score on the CaSUN was 74.2 (SD = 16.8). Among the five domains of the scale, the most frequently reported USCNs were in the information and medical care (F1) domain, with a score of 22.0 (SD = 5.6), followed by the life perspective domain at 18.7 (SD = 5.4). As for their spousal caregivers, the mean total score on the C-CaSPUN was 72.0 (SD = 15.5). Across its four domains, the most commonly reported USCNs fell within the relationship impact and life perspective (F1) domain, scoring 35.1 (SD = 9.1), while the least reported USCNs were in the quality-of-life domain, with a score of 5.8 (SD = 3.8). In terms of depression, the mean scores were 9.4 (SD = 4.3) for CRC patients and 8.8 (SD = 3.7) for their spousal caregivers.

Paired t-tests showed that CRC patients had significantly higher total USCNs scores (*t* = 2.155, *p* = 0.032) and depression scores (*t* = 2.356, *p* = 0.019) than their spousal caregivers. Detailed results are provided in Table 2.

**Table 2:** Comparative between-group analyses of study indicators.

Indicators	CRC patients (M ± SD)	CRC spousal caregivers (M ± SD)	*t*	*P*
USCNs				
C-CaSUN total scale	74.2 (16.8)		2.155	0.032
IAMC (P-F1)	22.0 (5.6)			
LP (P-F2)	18.7 (5.4)			
R (P-F3)	13.8 (4.9)			
CS (P-F4)	8.0 (4.9)			
QOL (P-F5)	11.7 (2.6)			
C-CaSPUN total scale		72.0 (15.5)		
RIALP (C-F1)		35.1 (9.1)		
IAHC (C-F2)		23.2 (4.8)		
QOL (C-F3)		5.8 (3.8)		
SC (C-F4)		7.9 (2.2)		
Depression	9.4 (4.3)	8.8 (3.7)	2.356	0.019

USCNs, unmet supportive care needs; CRC, colorectal cancer; M ± SD = Mean ± Standard deviation. C-CaSUN: the Chinese version of Cancer Survivors’ Unmet Needs Scale, IAMC (P-F1): information and medical care (P-F1), LP (P-F2): life perspective (P-F2), R (P-F3): relationship (P-F3), CS (P-F4): comprehensive support (P-F4), QOL (P-F5): quality of life (P-F5). C-CaSPUN: the Chinese version of Cancer Survivors’ Partners Unmet Needs measure, RIALP (C-F1): relationship impact and life perspective (C-F1), IAHC (C-F2): information and health care (C-F2), QOL (C-F3): quality of life (C-F3), SC (C-F4): survivorship care (C-F4). Statistical comparisons (*t* and *p*-values) were only conducted on the total USCNs and depression scores due to the structural differences in the sub-scales between patients and caregivers.

Preliminary bivariate Pearson correlation analyses were conducted among the primary study variables (Table 3). Regarding intra-personal associations, patients’ USCNs were significantly and positively correlated with their own depression (*r* = 0.327, *p* < 0.01), as were caregivers’ USCNs with their own depression (*r* = 0.253, *p* < 0.01). Regarding inter-personal (cross-over) associations, caregivers’ USCNs showed a significant positive correlation with patients’ depression (*r* = 0.250, *p* < 0.01), while the correlation between patients’ USCNs and caregivers’ depression was not statistically significant (*r* = 0.128, *p* > 0.05). Notably, strong positive correlations were observed between patients’ and caregivers’ USCNs (*r* = 0.599, *p* < 0.01) and between their respective depression levels (*r* = 0.539, *p* < 0.01). This high degree of non-independence within the dyadic data necessitated the use of a more rigorous dyadic analytic approach.

**Table 3:** Correlation between USCNs and depression in CRC dyads (r value,n = 200).

**Indicators**	**“r” with CRC patients USCNs**	**“r” with CRC spousal caregivers USCNs**	**“r” with CRC patients depression**	**“r” with CRC spousal caregivers depression**
CRC patients USCNs	1	**—**	—	—
CRC spousal caregivers USCNs	0.599**	1	—	—
CRC patients depression	0.327**	0.250**	1	—
CRC spousal caregivers depression	0.128	0.253**	0.539**	1

USCNs, unmet supportive care needs; CRC, colorectal cancer. Correlation coefficients (*r*) are shown; *p < 0.05 (two-tailed), **P < 0.01 (two-tailed); “—” indicates self-correlation (1.0) or redundant data.

### 3.3 The APIM of USCNs and depression

**Figure 1:**
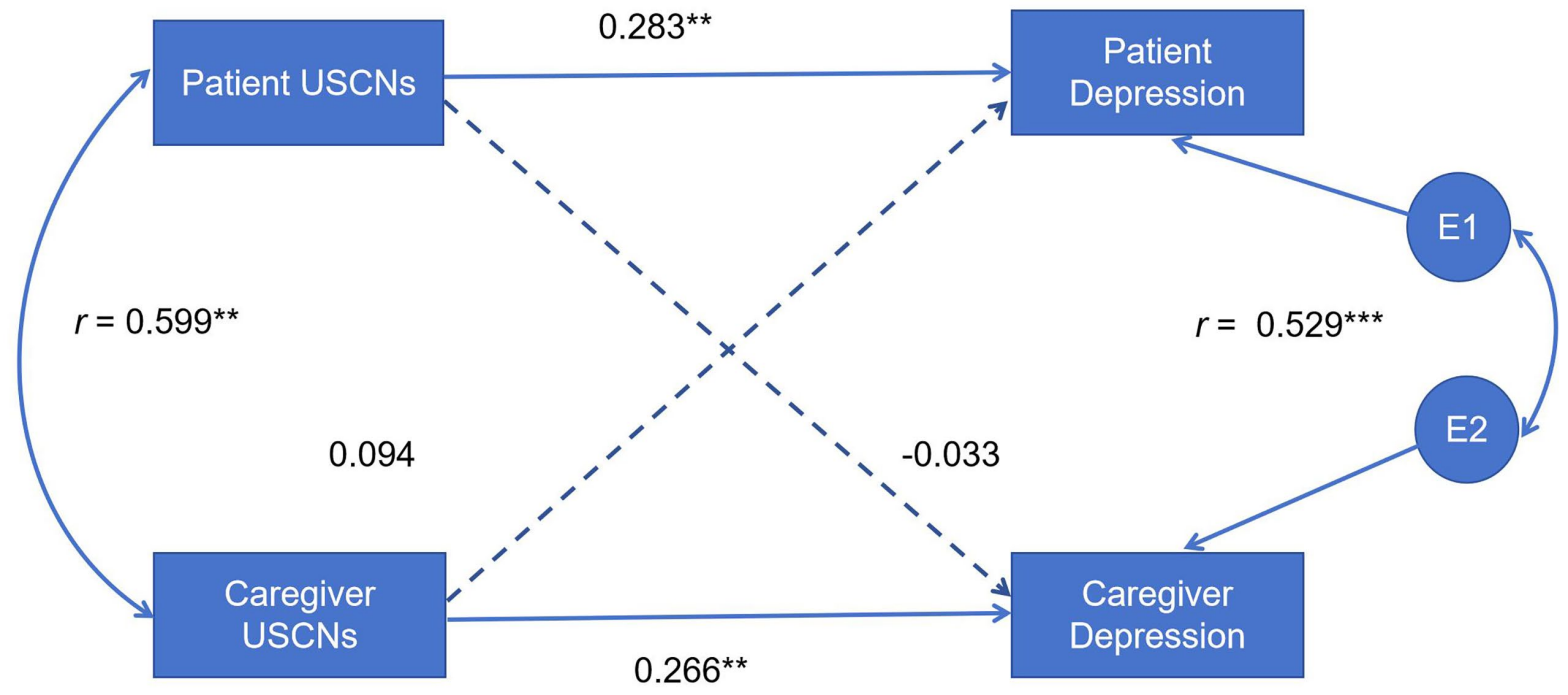
The actor-partner interdependence model (APIM) examining the dyadic effects of unmet supportive care needs (USCNSs) on depression. Standardized path coefficients (β) are presented on the straight arrows. Solid lines indicate statistically pathways, and dashed lines indicate non-significant pathways. The curved arrows represent the bivariate correlation (*r*) and the residual correlation partial *r. p* < 0.01, ***p < 0.001.

To account for the non-independence of dyadic data—highlighted by the strong correlation between patients’ and caregivers’ USCNs (*r* = 0.599, *p* < 0.01)—APIM was estimated using multilevel modeling. Diagnostic tests supported the APIM specification. The ICC for depression was 0.539 (*p* < 0.01), exceeding the 0.05 threshold and confirming significant dyadic interdependence. An overall test of distinguishability was significant [χ^2^ (4) = 10.976, *p* = 0.027], supporting the use of a distinguishable model for patients and spousal caregivers. Furthermore, the post-centering Variance Inflation Factor (VIF) was 1.56, indicating no severe multicollinearity, and residual plots confirmed that the assumptions of normality and homoscedasticity were met (Figure 1 and Table 4).

**Table 4:** APIM results for the predictive effects of USCNs on depression in CRC dyads.

**Indicators**	**Effect**	**Estimate (*b*)**	**95% CI**	***β***	***t* value**	***P* value**
Patient depression						
Intercept		2.502	[−0.411, 5.415]	-	1.68	0.093
Patient USCNs	Actor	0.070	[0.028, 0.112]	0.283	3.26	0.001**
Caregiver USCNs	Partner	0.023	[−0.022, 0.069]	0.094	1.01	0.313
Caregiver depression						
Intercept		4.594	[1.995, 7.194]	-	3.47	<0.001***
Caregiver USCNs	Actor	0.066	[0.026, 0.107]	0.266	3.19	0.002**
Patient USCNs	Partner	−0.008	[−0.045, 0.029]	−0.033	−0.42	0.672

USCNs: unmet supportive care needs; b: unstandardized coefficient; CI: confidence interval; β: standardized coefficient. **p < 0.01; ***p < 0.001.

The path analysis results revealed statistically significant but modest actor effects for both members of the dyad. Specifically, patients’ own USCNs significantly positively predicted their depression levels (*b* = 0.070, *β* = 0.283, *p* = 0.001), as did caregivers’ USCNs for their own depression (*b* = 0.066, *β* = 0.266, *p* = 0.002). This model explained 10.2 and 5.5% of the variance in depression for patients and caregivers, respectively.

However, no significant partner effects were found in either direction. Caregivers’ USCNs did not significantly predict patients’ depression (*b* = 0.023, *β* = 0.094, *p* = 0.313), and patients’ USCNs demonstrated no significant predictive effect on caregivers’ depression (*b* = −0.008, *β* = −0.033, *p* = 0.672). The discrepancy with the bivariate analysis likely reflects the difference between zero-order correlations and APIM estimates. Although caregivers’ USCNs were correlated with patients’ depression (*r* = 0.250), this association was no longer significant after patients’ own USCNs and dyadic interdependence were accounted for, suggesting that it reflected shared dyadic variance rather than a unique partner effect. After accounting for these predictors, a significant residual correlation between dyadic depression scores remained (partial *r* = 0.529, *p* < 0.001), indicating shared emotional variance beyond the modeled USCNs.

### 3.4 Qualitative findings: mechanisms underlying the APIM results

In line with the explanatory sequential design, the qualitative phase was used to contextualize the APIM findings, particularly why unmet needs significantly predicted individuals’ own depression but did not show significant partner effects. Through reflexive thematic analysis, we developed two interconnected dyadic themes that explain this pattern: internalization of unfulfilled needs and protective buffering with emotional compartmentalization.

#### 3.4.1 Theme 1: internalization of unfulfilled needs (explaining significant actor effects)

This theme contextualized the significant actor effects by showing how unmet needs were often processed as private cognitive and emotional burdens rather than openly shared within the dyad. Both patients and caregivers described keeping worries to themselves and managing distress alone, which helped explain why their own unmet needs were associated with their own depressive symptoms.

For patients, insufficient medical information and challenges in managing treatment side effects often trigger rumination and catastrophic thinking. They often chose to remain silent to avoid being a burden to their families. Hesitant to “bother busy doctors” (P7), many resorted to self-directed searching, inadvertently heightening their anxiety:

*“I always feel that just listening to the doctor isn’t enough, so I can’t help but search for information about this disease on my phone. But after reading it, I get even more worried. It feels like a heavy stone is pressing on my chest, making it hard to breathe.” (P8) The metaphor of a “heavy stone” illustrates how unmet informational needs were internalized as rumination and anxiety, helping to contextualize the significant patient actor effect observed in the APIM.*

Similarly, spousal caregivers internalized their unfulfilled support needs. Overwhelmed by the dual burdens of caregiving and financial strain, they often bore the psychological pressure independently:

*“I have no one around to confide in, so I just have to swallow all the bitterness into my own stomach… I feel like a spinning top that never stops, and my nerves are stretched so tight they could snap at any moment.” (C10) Similarly, the expression “swallowing bitterness” suggests that caregivers often kept their unmet support needs private, which is consistent with the significant caregiver actor effect.*

#### 3.4.2 Theme 2: protective buffering and emotional compartmentalization (explaining non-significant partner effects)

A prominent finding was the frequent use of *protective buffering*—a dyadic communication strategy where individuals intentionally concealed their unfulfilled needs and fears to shield their partners from additional distress. This emotional compartmentalization effectively severed the interpersonal transmission of psychological burden, contextualizing the non-significant partner effects observed in the APIM.

Caregivers frequently masked their emotional needs to present a facade of strength. As one caregiver articulated:

*“When she got the diagnosis… she just collapsed. To be honest, I was terrified too. But as a man, how could I possibly show that? I had to force myself to stay calm… How could I possibly pass my negative emotions on to her when she is already suffering so much?” (C8) By concealing his own distress, the caregiver limited the overt transmission of unmet needs to the patient, which helps contextualize the non-significant caregiver-to-patient partner effect.*

Furthermore, the fear of “saying the wrong thing” precipitated a communication avoidance. Caregivers suppressed their communication needs to avoid exacerbating the patient’s depression:

*“I actually really want to comfort her… But when it’s time to speak, I hesitate. I am terrified of saying the wrong thing and making her overthink. If my words make her feel worse, I would blame myself even more.” (C2) Here, the fear of causing additional emotional harm turned communication into self-censorship, restricting the direct sharing of unmet needs between partners.*

While protective buffering diminished the direct interpersonal prediction of depression, it inadvertently fostered a sense of isolation among patients, who remained sensitive to communication barriers:

*“Since the diagnosis, our communication has dropped significantly… I know he is afraid of saying the wrong thing and hurting me. But I really wish he would talk to me more, even just telling me what he is worrying about. I am not afraid to hear it; in fact, knowing his thoughts would make me feel more grounded.” (P4) These narratives suggest that the absence of statistical partner effects may reflect emotional containment and protective buffering, rather than a lack of concern between partners. Thus, protective buffering may have reduced the overt sharing of unmet needs while leaving both partners to cope with distress in a more isolated manner.*

#### 3.4.3 Summary of mixed-methods integration

In summary, the qualitative themes contextualize the APIM findings: the non-significant partner effects do not reflect true dyadic emotional independence, but rather result from a systematic pattern of protective buffering.

By actively concealing their unmet needs to protect one another, both dyad members inadvertently isolate themselves, thereby amplifying their intrapersonal distress (the significant actor effects). As shown in Table 5, we used a joint display to formally integrate the findings.

**Table 5:** Joint display of quantitative findings and qualitative results.

**Quantitative findings**	**Qualitative results**	**Integrated interpretation**
Significant Actor Effects: Participants’ USCNs predicted their own depression (*β* = 0.283/0.266, *p* < 0.01).	Internalization of needs: Participants processed stress as a private burden. “A heavy stone pressing on my chest” (P8). “Swallow all the bitterness” (C10).	Internalization pathway: Participants’ own unmet needs may contribute to depressive symptoms through private rumination and emotional burden.
Non-significant Partner Effects: Needs did not significantly cross over between partners (*p* > 0.05).	Protective buffering: Individuals intentionally concealed their needs and fears to shield the other. Maintain a “facade of strength” (C8). “Terrified of saying the wrong thing” (C2).	Protective buffering pathway: Concealment of needs may limit overt emotional sharing, helping to contextualize the non-significant partner effects.
USCNs Profile Divergence: Patients reported higher Information needs; Caregivers reported higher Relationship needs.	Cultural Roles: Patients struggled with “being kept in the dark,” while caregivers suppressed needs due to “family-first” norms and simultaneously yearn for emotional and interpersonal support. “Just listening to the doctor is not enough” (P8). “I have no one around to confide in” (C10).	Role-specific constraints: Cultural practices like “protective truth-telling” shape distinct USCNs patterns for patients and caregivers.
Strong Residual Correlation: A significant correlation remained between partners’ depression (*r* = 0.529, *p* < 0.001).	Shared Existential Threat: Despite communication blocks, partners sensed each other’s distress and desired “grounded” connection. Need for thoughts to feel “more grounded” (P4).	Shared emotional context: Although specific unmet needs were not directly transmitted as partner effects, dyads remained emotionally connected through the shared threat of cancer.

USCNs: unmet supportive care needs; β: standardized coefficient.

## 4 Discussion

Our mixed-methods study, guided by the DCT, explored the dyadic interrelationships between USCNs and depression among CRC patient-spousal caregiver dyads. The findings not only clarify the current status of USCNs and depression in this population but also reveal the underlying psychosocial mechanisms, providing valuable insights for clinical practice.

### 4.1 Current status of USCNs and depression in CRC patient-spousal caregiver dyads

Quantitative findings showed that CRC patients reported significantly higher levels of both depression and USCNs compared to their spousal caregivers. The cultural environment could account for this discrepancy (Kim et al., 2008). To put patient care first, spousal caregivers, especially spouses who have been impacted by the conventional norm of “family first,” may suppress their own needs and emotions (Northouse et al., 2000; Emslie et al., 2009). Additionally, most of the patients in this study were undergoing chemotherapy or surgery, which exacerbated their depressive symptoms (Han et al., 2020). The documented phenomena of CRC dyads having depression scores beyond the cutoff of 8 are partly explained by the strong psychological relationship between spouses. While traditional literature often assumes this shared distress is directly transmitted between partners (Cheng et al., 2022; Li and Loke, 2014), this assumption necessitates a more rigorous dyadic exploration, which we address through our integrated APIM findings.

Additionally, the study discovered variations between CRC dyads in specific USCN domains. The Information and Medical Care domain accounted for the majority of patients’ most urgent USCNs. This may be connected to the widespread cultural practice in Chinese families of protecting patients by keeping them in the dark about their illnesses, which ironically makes patients want clear and authoritative medical information to reclaim control (Wu et al., 2022). In contrast, spousal caregivers had the highest USCNs in the Relationship Impact and Life Perspective domain, which covered finding purpose in life, coping with changes in the marital relationship, and seeking emotional support (Li et al., 2020a). The majority of caregivers in this study were female, 57% reported a decline in their marital relationship, and 55% had been providing care for less than 6 months, a time that is frequently characterized by role uncertainty and challenges adjusting to new relationships (Northouse et al., 2000; Pape et al., 2022). As a result, they are especially in need of emotional support and relationship restoration (Li et al., 2020a).

### 4.2 The dyadic paradox: mechanisms underlying the APIM results

To move beyond descriptive trends, our rigorous APIM analysis revealed a distinct dyadic pattern: strong actor effects coupled with non-significant partner effects. This indicates that an individual’s depressive symptoms are primarily driven by their own unfulfilled needs rather than a direct transmission of their partner’s needs. This “dyadic paradox”—where couples share deep distress but do not directly transmit their specific unmet needs to one another—can be systematically understood through the lens of DCT.

#### 4.2.1 The internalization of distress and robust actor effects

According to DCT, how individuals cognitively appraise and manage their own stress within a dyad directly impacts their wellbeing (Bodenmann et al., 2006). For both patients and caregivers, the significant actor effects are largely rooted in the internalization of their specific unfulfilled needs. As previously noted, patients grapple with the physiological traumas of cancer and the anxiety of information deprivation (Wu et al., 2022). When these needs remain unmet, they internalize the distress, directly fueling their own depression (Grov et al., 2005). Furthermore, patients frequently attribute their spouses’ fatigue to their own illness, experiencing profound guilt (Chen et al., 2023), which acts as a strong internal predictor of depressive symptoms (Chen et al., 2025). Conversely, caregivers internalize the exhaustion of sudden role transitions (Pape et al., 2022) and their suppressed need for emotional support (Hodgkinson et al., 2007), validating the caregiver actor effect. While statistically significant, these actor effects explained a modest proportion of variance (*R*^2^ = 5.5–10.2%), indicating that USCNs accounted for only part of the variability in depressive symptoms.

#### 4.2.2 Negative dyadic coping and the non-significant partner effects

While previous studies might predict a cross-over of distress (Cheng et al., 2022), the APIM demonstrated that direct partner effects were not statistically significant. It is important to clarify that a non-significant *p*-value does not inherently prove the absence of interpersonal influence; rather, it indicates that a predictive relationship was not detectable within our specific sample.

Viewed through the framework of DCT, our qualitative findings help explain this statistical absence as a clear manifestation of negative dyadic coping—specifically, the extensive use of protective buffering (Bodenmann et al., 2006). In order to lessen emotional investment and shield the patient, caregivers often employ task-oriented coping strategies within this protective buffering framework, redefining exhausting care responsibilities as controllable duties while deliberately hiding their own vulnerability (Yan et al., 2024). Conversely, patients conceal their fears of recurrence and informational anxieties to avoid burdening their exhausted caregivers (Sun et al., 2025). Ultimately, this mutual protective buffering acts as a “double-edged sword.” On one hand, it acts as an effective emotional firewall, cutting off the direct interpersonal transmission of specific unfulfilled needs. These communication barriers provide a psychosocial context for the non-significant partner effects observed in the APIM. On the other hand, it disrupts authentic dyadic communication, forcing both partners into a state of increased interpersonal isolation.

Despite the absence of direct partner effects, the APIM revealed a significant residual correlation (partial *r* = 0.529) between the dyad’s depression scores. This underscores that while protective buffering mitigates the direct interpersonal transmission of specific unmet needs, both partners remain emotionally synchronized by the overarching, shared existential threat of cancer. Additionally, methodological constraints should be considered. Although the sample size (n = 200 dyads) met the a priori requirement for the primary analyses, it may still have had limited power to detect very small partner effects (Ledermann et al., 2022). Moreover, the CaSUN and CaSPUN are role-specific instruments designed to assess individual unmet needs among patients and partners, respectively; therefore, they may be less sensitive to dynamic cross-partner transmission of unmet needs or distress than couple-specific measures.

### 4.3 Limitations

The interpretability and generalizability of our findings are subject to several considerations. First, the reliance on convenience sampling limits the generalizability of our findings. The sample was drawn from a single hospital, which may not fully represent the demographic and clinical diversity of the broader CRC population. Second, the cross-sectional design precludes causal inference and cannot capture the temporal dynamics of dyadic interactions. While the sample included patients at various times since diagnosis, future longitudinal studies are warranted to stratify analyses by distinct treatment phases and track these interdependent trajectories over time (So et al., 2019). Third, the observed phenomena, such as protective buffering and information concealment, are deeply rooted in Chinese cultural norms (e.g., Familism) (Qiu et al., 2025a); thus, the generalizability of these dyadic interaction patterns to Western populations requires further cross-cultural validation. Fourth, methodological constraints must be noted. The sample size and the individual-centric design of the assessment tools may have limited the detection of subtle partner effects. Furthermore, while we utilized Table 5 to integrate our mixed-methods findings, the inherent complexity of fully merging qualitative and quantitative data remains a challenge that may affect the depth of data interpretation Rather than openly disclosing distress, patients and caregivers may withhold emoti. Finally, the reliance on self-reported questionnaires may introduce social desirability bias; specifically, the culturally-driven “protective buffering” likely prompted participants to under-report their USCNs or depressive symptoms to shield their partners or maintain family harmony, potentially leading to an underestimation of these variables.

### 4.4 Implications and conclusions

In conclusion, this explanatory sequential mixed-methods study clarifies the complex interdependence between USCNs and depression in CRC dyads. Our APIM analysis revealed an important dyadic dynamic: while significant actor effects demonstrated that unfulfilled needs predict intrapersonal depression, the absence of partner effects indicated no direct interpersonal transmission of these specific needs. The qualitative findings explained this phenomenon, revealing that couples frequently employ “protective buffering” to shield one another. However, this well-intentioned concealment restricts authentic communication, leaving both partners to navigate their distress independently.

Based on these patterns, future interventions could focus on reducing the burden of protective concealment rather than simply encouraging general communication. The significant actor effects suggest that individual-level support remains necessary, while the qualitative finding of protective buffering indicates the need for couple-based strategies that help partners transform concealment into shared problem-solving and emotional disclosure. Providing medical information to both partners simultaneously may also help reduce protective truth-telling and support more transparent dyadic coping. Overall, oncological care could benefit from treating the dyad as an interdependent unit while tailoring support to both individual distress and couple-level communication barriers.

## Data Availability

The raw data supporting the conclusions of this article will be made available by the authors, without undue reservation.
